# Synbiotic combination of *Bifidobacterium longum* BB536 and lactulose improves the presenteeism of healthy adults associated with aromatic lactic acids - A single-arm, open-label study

**DOI:** 10.1080/29933935.2025.2490092

**Published:** 2025-04-10

**Authors:** Riko Mishima, Ryuta Ejima, Satoshi Arai, Ayako Horigome, Eri Mitsuyama, Hiroki Kaneko, Kana Yamaguchi, Keita Kamezaki, Yuka Togashi, Kiyoshi Nakamura, Noriyuki Iwabuchi, Miyuki Tanaka, Toshitaka Odamaki, Shinji Fukuda, Yuji Naito

**Affiliations:** a Innovative Research Institute, Morinaga Milk Industry Co., Ltd., Zama, Kanagawa, Japan; b Metagen, Inc., Tsuruoka, Yamagata, Japan; c Medical Corporation Hiroaki Kunitachi Sakura Hospital, Kunitachi, Tokyo, Japan; d Institute for Advanced Biosciences, Keio University, Tsuruoka-City, Yamagata, Japan; e Gut Environmental Design Group, Kanagawa Institute of Industrial Science and Technology, Ebina, Kanagawa, Japan; f Transborder Medical Research Center, University of Tsukuba, Tsukuba-City, Ibaraki, Japan; g Innovative Microbiome Therapy Research Center, Juntendo University Graduate School of Medicine, Bunkyo-Ku, Tokyo, Japan; h Department of Human Immunology and Nutrition Science, Kyoto Prefectural University of Medicine, Kyoto, Japan

**Keywords:** Synbiotics, gut microbiota, QOL, presenteeism, aromatic lactic acids

## Abstract

Gut microbiota and their metabolites are crucial for metabolic regulation, immunomodulation, and brain and mental functions via the brain-gut axis. Synbiotics offer an effective strategy for improving gut health by modulating gut microbiota, leading to improved physical and mental health. In the present study, an exploratory investigation into the effects of synbiotic yogurt on physical and mental health was conducted. Healthy adults consumed fermented milk containing *Bifidobacterium longum* subsp. *longum* BB536 (2 billion colony-forming units) and lactulose (4 g) daily for four weeks. Post-intervention, we observed an increase in bowel movement scores and improved life quality metrics, including both the WHO-HPQ absolute and relative presenteeism and the WHO-SUBI well-being score. In addition, significant increases in *Bifidobacterium*, butyric acid-producing bacteria, and various metabolites were observed. Furthermore, the well-being score correlated positively with *Fusicatenibacter* and short-chain fatty acids, whereas absolute presenteeism correlated positively with *Bifidobacterium*, aromatic lactic acids (ALAs), and homocysteine, and negatively with urocanic acid. Additionally, our analysis revealed that the presence of *Bifidobacterium* and aromatic amino acids in the gut influenced ALA production. The results indicate that synbiotic yogurt may improve bowel movement and life satisfaction, highlighting the importance of gut microbiota-derived metabolites in promoting overall well-being.

## Graphical abstract

**Figure:**
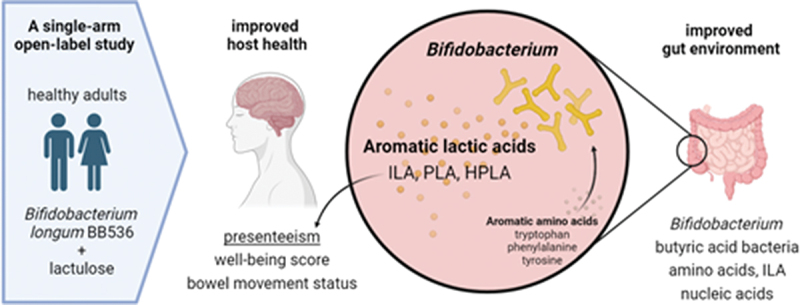

## Introduction

Gut microbiota, a complex community of microorganisms residing in the digestive tract, has been associated with myriad health outcomes. They play crucial roles in maintaining physical health by influencing nutrient digestion and absorption,^52^ metabolic processes,^55^ and the immune system.^57^ Furthermore, the relationship between the gut microbiota and mental health has been receiving increasing attention.^7^ Probiotic supplementation^53^ and tryptophan metabolites derived from the gut microbiota^45^ can positively affect psychological disorders, such as anxiety, depression, and autism by affecting the brain-gut axis (BGA). Therefore, the gut microbiota is intricately associated with both physical and mental well-being.

These physical and mental health conditions permeate various facets of life, including daily activities and workplace performance. For instance, presenteeism, a phenomenon where employees are physically present at work but suffer from reduced performance and productivity owing to physical or mental health issues, is becoming increasingly prevalent.^13^ This issue is typically quantified using methods such as the WHO-HPQ absolute presenteeism (a subjective evaluation of one’s performance) and the WHO-HPQ relative presenteeism (an evaluation of one’s performance compared to others). In Japan, presenteeism results in an annual economic loss of $3055 per person, significantly surpassing the losses caused by absenteeism ($520) and medical/pharmaceutical expenses ($1165).^28^ Gut microbiota could offer a novel approach to address quality of life (QOL) issues such as presenteeism. Several studies have highlighted the efficacy of probiotics for improving work performance, including *Lactococcus lactis* subsp. *lactis* ^19^, *Lacticaseibacillus paracasei* ^16^, *Pediococcus acidilactici*, and *Lactiplantibacillus plantarum*.^39^ However, these studies have not elucidated the relationship between the gut microbiota, metabolites, and presenteeism.

*Bifidobacterium longum* subsp. *longum* BB536 (hereafter referred to as *B. longum* BB536) is a versatile probiotic that has been safely utilized for over 50 years, offering a range of health benefits, such as improved bowel movements and immune system modulation.^50^ Furthermore, bifidobacterial species mainly present in the infant gut, such as *B. longum* BB536, produce substantial amounts of aromatic lactic acid (ALAs) from tryptophan, phenylalanine, and tyrosine, and their related metabolites.^22,37^ Synbiotic combinations with lactulose, an indigestible milk oligosaccharide, have been suggested to enhance the production of these metabolites in vitro study.^12^ These bacteria-driven metabolites have the potential to enhance QOL through BGA by activating the aryl hydrocarbon receptor (AhR) and hydroxycarboxylic acid receptor 3 (HCA3).^22,36^

Therefore, in this study, we investigated the effects of a synbiotic product containing *B. longum* BB536 and lactulose on physical and mental health. We examined the changes in defecation status, QOL, gut microbiota, and metabolites before and after the consumption of synbiotic yogurt. Our findings suggest that the synbiotic modifies the profile of the gut microbiota and metabolome, potentially contributing to improvements in defecation status and QOL.

## Materials and methods

### Clinical trial design

This single-arm, open-label study was conducted at Metagen, Inc. (Tsuruoka, Japan) from August to November 2023. This clinical trial was approved by the Ethics Committee of the Japan Conference of Clinical Research (registration number: 2023–013, date of board: 2023. 4. 18). The study protocol was registered with the University Hospital Medical Information Network (UMIN000051912). The study adhered to the Declaration of Helsinki (revised in 2013, Fortaleza) and the Ethical Guidelines for Life Science and Medical Research Involving Human Subjects (Notification No. 1, 2021, issued by the Ministry of Education, Culture, Sports, Science and Technology, the Ministry of Health, Labour and Welfare, and the Ministry of Economy, Trade and Industry). Before the start of the study, all participants were thoroughly informed of its purpose and procedures. Written informed consent was obtained from all the participants.

The study design included a two-week pre-observation period followed by a 28-d intervention period. During the intervention period, the participants were instructed to consume 100 g of synbiotic yogurt once daily. Throughout both the pre-observation and intervention phases, the participants maintained a daily log recording of their defecation, health status, and intake of medication and supplements. Additionally, the QOL of the participants was assessed via questionnaires on intervention days 0 and 28. Stool samples were collected on days 0, 3, and 28. The sample size was determined based on the maximum number of cases that could be performed at the institution.

### Participants

Healthy adults aged 18–74 years were recruited for this study. Participants were excluded if they fulfilled any of the following exclusion criteria: (1) regular use of medications such as laxatives, intestinal regulators, or other drugs that affect bowel movements; (2) regular consumption of foods or supplements containing lactic acid bacteria, bifidobacteria, oligosaccharides, or dietary fiber; (3) presence or medical history of diseases related to the liver, kidney, heart, lung, gastrointestinal tract, blood, endocrine system, or metabolism; (4) severe drug or food allergies, or a history thereof; (5) milk allergy or lactose intolerance; (6) pregnancy, lactation, or possibility of pregnancy during the study; (7) participation in other drug or food trials within the past month, or intention to participate in such trials; (8) any other conditions deemed as inappropriate for participation by the principal investigator based on the participant’s background, physical findings, examination, or physiological tests.

**Figure 1.**
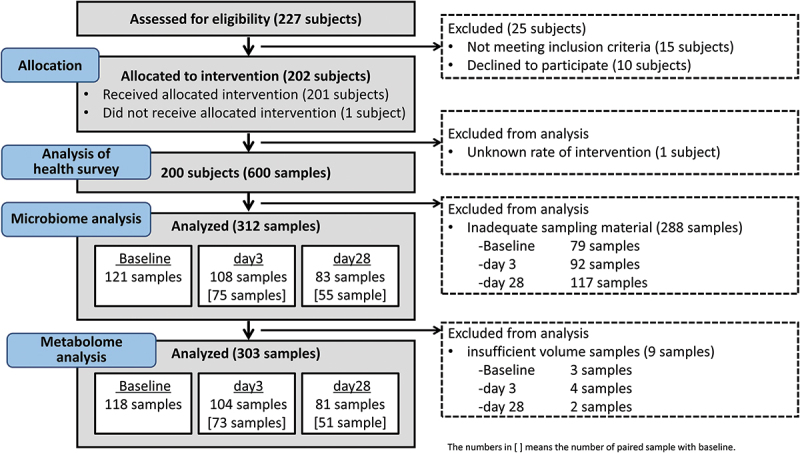
Clinical trial flowchart.

Of the 227 individuals who provided consent, 202 met the inclusion criteria and were enrolled. One participant withdrew from the study during the pre-observation period. Another participant whose synbiotic yogurt consumption rate was unknown was excluded from the analysis. Of the fecal samples collected from 200 participants, 312 were used for microbiota and metabolite analysis, excluding those that were not adequately dried owing to an excess amount of sample relative to the silica gel in the MG kit (Metagen, Inc., Tsuruoka, Japan) (Figure 1, Supplementary Table S1).

### Questionnaire

The participants maintained a diary documenting their defecation habits, including stool frequency, volume, and form. Stool frequency was calculated as the number of times per week. To estimate stool volume, participants were asked to compare their stool to the size of a medium-sized chicken egg (4 × 5 cm), with each “egg” equivalent to 60 g of feces. Stool form was classified on a scale of 1 (hardest) to 7 (softest) using the Bristol Stool Form Scale (BSFS), and the average BSFS score per time was calculated. The baseline value was determined using data from one week before the intervention, whereas the value during the intervention was computed using data from the fourth week post-intervention (W4).

Additionally, we compared various QOL scores pre- and post-intervention.

Well-being was assessed using the Japanese version of the Subjective Well-Being Inventory (SUBI).^41^ The SUBI comprises 19 and 21 questions related to well-being and ill-being, respectively. Well- or ill-being scores were calculated from the total score for each question. A high well-being score indicates high levels of positive emotions, such as happiness and self-confidence, whereas a high ill-being score indicates low levels of negative emotions, such as disappointment and social rejection.

Work performance was assessed using the Japanese version of the World Health Organization Health and Work Performance Questionnaire (WHO-HPQ).^15^ Work performance was scored using the following WHO-HPQ parameters: absolute presenteeism, relative presenteeism, absolute absenteeism, and relative absenteeism.

Presenteeism is defined as reduced work performance owing to physical or mental health problems at work, whereas absenteeism refers to an absence from work because of health problems.

- WHO-HPQ absolute presenteeism: self-assessed work performance
- WHO-HPQ relative presenteeism: self-assessed work performance compared with the average performance of others
- WHO-HPQ absolute absenteeism: expected working hours minus actual working hours
- WHO-HPQ relative absenteeism: (expected working hours minus actual working hours) divided by expected working hours

### Supplementation

Synbiotic liquid yogurt was supplied by Morinaga Milk Industry (Tokyo, Japan). Each 100 g serving of yogurt contained 2 billion CFUs of *B. longum* BB536 and 4 g of lactulose. The participants were instructed to consume 100 g of yogurt daily during the 28-d intervention period.

### Sample collection

Fecal samples were collected on intervention days 0, 3, and 28, using an MG kit (Metagen, Inc., Tsuruoka, Japan). This kit rapidly dries feces using silica gel, thereby preserving microbiota and metabolome profiles. The dried samples were transferred into a screw-cap tube containing four 3-mm zirconia beads and crushed using a TissueLyser III (Qiagen, Venlo, The Netherlands) for 10 min at 25 hz. The powdered samples were stored at −80°C until further processing.

### Sample preparation

The samples were prepared according to the protocols described in Supplementary Table S2. Briefly, approximately 10 mg (±0.5 mg) of fecal samples were suspended in 300 μL of solution. These suspensions were further disrupted with 0.1 mm zirconia/silica beads by vigorous shaking (25 hz, 10 min) using a TissueLyser III and centrifuged at 20,380 × *g* for 5 min. The supernatants were then transferred into microcentrifuge filters and centrifuged at 9,100 × *g* for 5 min. The filtered samples were then transferred into ultrafiltration filters that were previously washed with 200 μL LC-MS-grade solution using centrifugation at 9,100 × *g* for 3 h. The filtered samples were stored at −80°C until analysis.

### HPLC

Ammonia and organic acids were determined by HPLC. HPLC analysis was performed using a Nexera X2 system (Shimadzu, Kyoto, Japan) equipped with two LC-40 D pumps, a DGU-405 degasser, a SIL-40C autosampler, a CTO-40C column oven, a CBM-40 control module, and a CDD-10A VP conductivity detector. The chemicals used for HPLC analysis are listed in Supplementary Table S3. HPLC measurements were conducted following the parameters summarized in Supplementary Tables S3 and S4.

### LC-MS/MS

Metabolites, excluding ammonia and organic acids, were analyzed using LC-MS/MS. The LC-MS/MS analysis was conducted using a Nexera X2 system (Shimadzu, Kyoto, Japan) equipped with two LC-40D XR pumps, a DGU-405 degasser, a SIL-40C XR autosampler, a CTO-40C column oven, and a CBM-40 control module. The system was coupled to an LC-MS-8045 triple-quadrupole mass spectrometer (Shimadzu, Kyoto, Japan). All chemicals used for the target analysis, including internal standards, were sourced from various suppliers (Supplementary Table S5). LC-MS-grade water, methanol, acetonitrile, and formic acid were obtained from FUJIFILM Wako Pure Chemical Corporation. LC-MS/MS measurements were performed according to the parameters described in Supplementary Tables S4 and S5.

### 16S rRNA gene amplicon analysis

Bacterial DNA was extracted from 10 mg (±0.5 mg) of feces and amplified as previously described.^27^ The V3-V4 region of the bacterial 16S rRNA gene was paired-end sequenced using the Illumina NextSeq 1000 platform with the NextSeq 1000/2000 P1 Reagent kit (600 cycles) (Illumina, San Diego, CA, USA).

The sequences were analyzed using QIIME2 software (version 2022.8).^2^ The demultiplexed reads were processed by filtering, denoising, merging, chimera removal, and generation of amplicon sequence variants (ASVs) using DADA2.^4^ The ASVs were assigned to a taxonomy based on the Greengeens2 database (version 2022.10). An average of 24,134 ± 18,286 reads were obtained per sample.

### Statistical analysis

Statistical analyses were conducted using R software (version 4.3.1). Differences within and between groups were evaluated using the Wilcoxon signed-rank and Mann – Whitney U tests, respectively. Multiple testing corrections were applied using the Benjamini – Hochberg correction. Spearman’s rank correlation analysis and the partial Spearman’s rank correlation were conducted to assess the relationships between the variables. The ALDEx2 (ANOVA-Like Differential Expression Analysis version 2) was used to identify genera with significant differences compared to the baseline. Genera with a relative abundance greater than 0.1% were analyzed. The analysis involved the calculation of the centered log-ratio transformation abundance. Networks of the gut microbiota, metabolites, and QOL were visualized using Cytoscape (version 3.10.1). A *p*-value <0.05 was considered statistically significant.

### Accession number of bacterial 16S rRNA gene sequences

Raw sequence data were deposited in the DNA Data Bank of Japan (DDBJ) under BioProject no. PRJDB18853. This project includes links and access to fecal sample data (SAMD00821023 to SAMD00821334).

## Results

### Participant characteristics

A total of 200 healthy adults were enrolled in this study. The baseline characteristics of the participants are summarized in Table 1. The cohort comprised 60 men and 140 women, with an average age of 50.6 ± 11.8 years and an average BMI of 21.4 ± 3.1 kg/m^2^.

**Table 1.** Baseline characteristics of the participants.

	*n* = 200
Age (years)	50.62 ± 11.80
Gender (man/woman)	60/140
BMI	21.40 ± 3.11
Regular exercise habit (yes/no)	103/97
Occupation	
Employed	112
Self-employed	8
Medical professional	1
Part-time worker	29
Homemaker	29
Student	1
Unemployed	20
Occupation Involving Night Shifts (yes/no)	9/191
Days worked/week	
≤1 days	39
2–3 days	25
4–5 days	125
≥6 days	11
Number of People in Household	1.57 ± 1.24
Marital Status (yes/no)	131/69
Living with Spouse (yes/no)^a^	126/5
Presence of Children (yes/no)	128/72
Living with Children (yes/no)^b^	78/50

^a^Married individuals only.

^b^Individuals with children only.

Continuous values are represented as mean ± S.D.

### Safety assessment

One serious adverse event, acute glaucoma, was reported during the study period. However, this event was not attributed to the consumption of the test foods, based on an evaluation by the principal investigator.

### Bowel movement and QOL assessment

**Figure 2.**
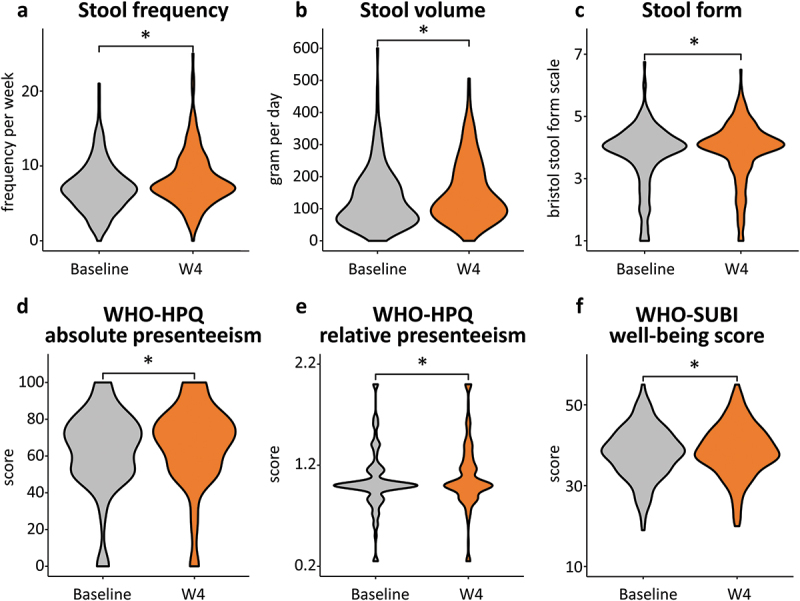
Pre- and post-intervention changes in bowel movement status and QOL scores with synbiotic yogurt. Violin plots depict (a) stool frequency, (b) stool volume, (c) stool form, (d) WHO-HPQ absolute presenteeism, (e) WHO-HPQ relative presenteeism, and (f) WHO-SUBI well-being score. “Baseline” represents the average over the week before the start of the intervention, and “W4” represents the average during the fourth week of the intervention. Differences were compared using the Wilcoxon signed-rank test (* *p* < 0.05).

We first examined bowel movement status pre- and post-intervention. A significant enhancement in stool frequency, volume, and form score was observed during the fourth week of the intervention (W4) relative to baseline (Wilcoxon signed-rank test, *p* < 0.05) (Figure 2a,b,c and Supplementary Table S6).

Subsequently, we assessed participants’ QOL. Notably, both absolute and relative presenteeism scores, along with the well-being score, demonstrated a significant increase during W4 relative to baseline (Wilcoxon signed-rank test, *p* < 0.05) (Figure 2d,e,f and Supplementary Table S6). In contrast, no significant alteration was noted in the scores for absolute and relative absenteeism or the WHO-SUBI ill-being score (Figure S1a, S1b, S1c and Supplementary Table S6). Furthermore, no differences in outcomes were observed between sexes or across different age groups. (Figure S2 and S3)

A significant positive correlation was observed between absolute presenteeism and stool frequency (Spearman’s correlation analysis, *rho* = 0.16, *p* < 0.05) (Table 2). However, no significant correlation was detected between absolute presenteeism and either stool volume or form or between relative presenteeism, well-being score, or bowel movement status (Table 2).

**Table 2.** Spearman correlation analysis between QOL scores and bowel movement status.

	Stool frequency		Stool amount		Stool form	
	*rho*	*p*	*rho*	*p*	*rho*	*p*
WHO-HPQ Absolute presenteeism score	0.16	0.02	0.12	0.08	0.06	0.40
WHO-HPQ Relative presenteeism score	0.01	0.92	0.07	0.31	−0.03	0.64
WHO-SUBI well-being score	0.07	0.36	0.09	0.23	0.005	0.95

### Gut microbiota and metabolome analysis

**Figure 3.**
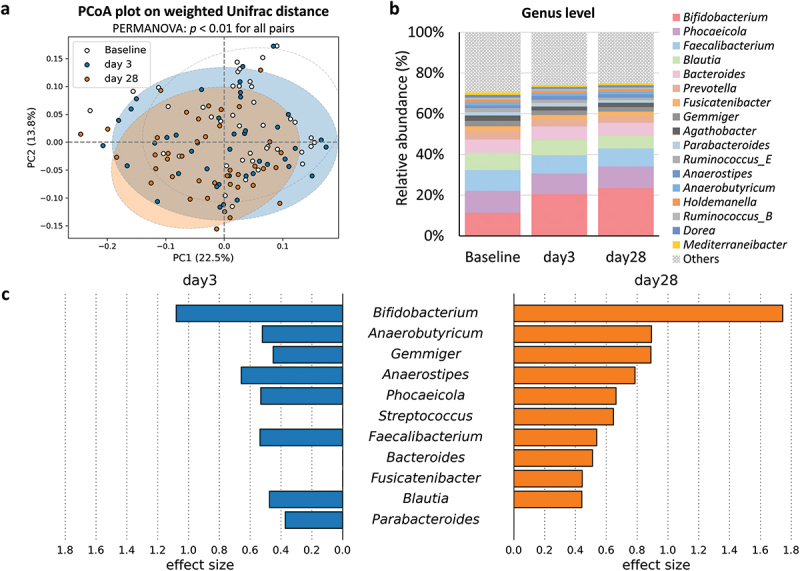
Alterations in gut microbiota during synbiotic yogurt administration. (a) Beta-diversity at baseline (white), day 3 (blue), and day 28 (orange) were assessed using principal coordinate analysis based on weighted Unifrac distance with 95% confidence intervals. Permanova shows significant differences between all time points (FDR-corrected *p* < 0.01). (b) The stacked bar plot illustrates the composition of gut microbiota at the genus level. Only taxa with an average relative abundance exceeding 0.1% are presented. (c) The butterfly chart displays that the ALDEx2 effect size of genera exhibited a significant increase (FDR-corrected *p* < 0.05) at day 3 (blue) and day 28 (orange) compared to the baseline. “Baseline”, “day 3” and “day 28” represent the day before the start of the intervention, and three and 28 d after the start of the intervention, respectively.

We compared the gut microbiota at baseline (pre-intervention) with those at short-(day 3) and medium-term (day 28) post-intervention (Figure 3a,b). Significant alterations in the gut microbiota were observed as early as day 3 (PEMANOVA, FDR-corrected *p* < 0.01 for all pairs). Among the 88　genera, *Bifidobacterium* exhibited the most significant changes (ALDEx2, FDR-corrected, *p* < 0.05, effect size = 1.08). *Phocaeicola*, *Blautia*, *Parabacteroides*, and several butyric acid-producing bacteria, including *Anaerobutyricum*, *Gemmiger*, *Anaerostipes*, and *Faecalibacterium*, also showed a significant increase on day 3 compared to baseline (ALDEx2, FDR-corrected *p* < 0.05) (Figure 3c, Supplementary Table S7). On day 28, in addition to the aforementioned seven genera (excluding *Parabacteroides*), *Streptococcus*, *Bacteroides*, and *Fusicatenibacter* were significantly increased (ALDEx2, FDR-corrected *p* < 0.05) (Figure 3c, Supplementary Table S7).

**Figure 4.**
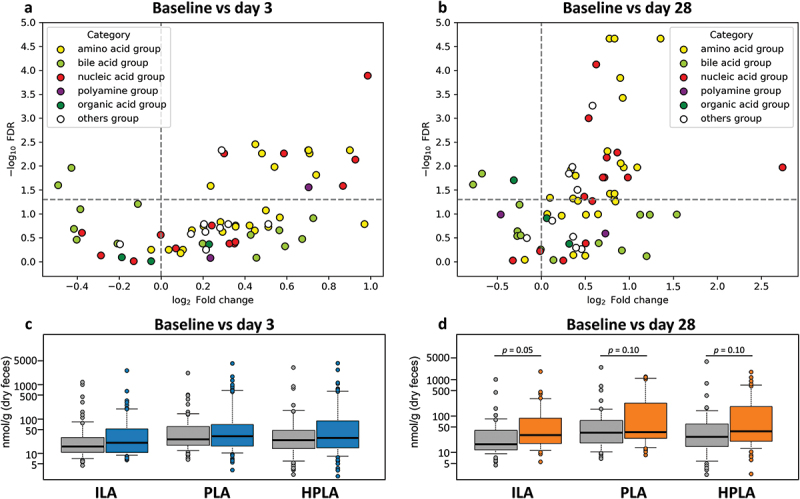
Metabolite changes during synbiotic yogurt administration. The volcano plots display the alteration of metabolites at (a) day 3 (*n* = 73) and (b) day 28 (*n* = 51). The y-axis represents the – log10 FDR-corrected *p*-values, while the x-axis shows the log2 fold change values. The color of the dots corresponds to the category of each metabolite as described in Supplementary Table S8. The boxplots describe the comparison of ALA concentrations between baseline vs day 3 (*n* = 73) and day 28 (*n* = 51). The p-values were calculated using the Wilcoxon signed-rank test and were adjusted using the false discovery rate (FDR).

We compared 67 fecal metabolites on days 3 and 28 (Figure 4a,b Supplementary Table S8). On day 3, 16 metabolites, including nine amino acid-group metabolites, five nucleic acid-group metabolites, one polyamine-group metabolite and one from the others-group, showed a significant increase, whereas two bile acid-group metabolites decreased (Wilcoxon signed-rank test, FDR-corrected *p* < 0.05) (Figure 4a). On day 28, 29 metabolites, including 16 amino acid-group metabolites, nine nucleic acid-group metabolites, and four others-group metabolites, showed significant increases, whereas three metabolites, including two bile acid-group metabolites and 1 organic acid-group metabolite, showed significant decreases (Wilcoxon signed-rank test, FDR-corrected *p* < 0.05) (Figure 4b).

A remarkable increase in several amino acid group metabolites was observed on day 28, including a significant increase in indole lactic acid (ILA), a tryptophan metabolite (Figure 4c,d). In addition, the levels of many nucleic acid group metabolites increased. The most significant changes were detected for adenine, along with adenosine, which are composed of ribose and adenine.

### Gut microbiota and metabolites correlated with QOL

**Figure 5.**
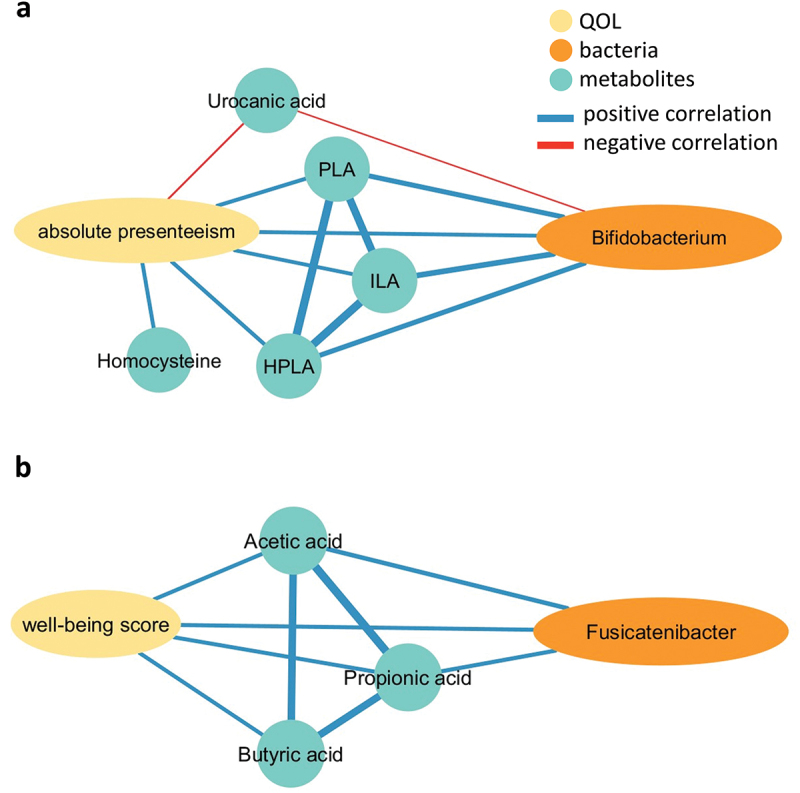
Correlations among gut microbiota, metabolites, and QOLs. The network was constructed based on Spearman rank analysis. Gut bacteria and metabolites, which are significantly correlated with (a) absolute presenteeism or (b) well-being score, are displayed. The edge color represents positive correlation (blue) and negative correlation (red), whereas the node color indicates QOL (yellow), bacterial genera (orange), and metabolites (green).

We conducted a network analysis based on Spearman’s correlation to profile the gut microbiota and metabolites associated with QOL. Our analysis revealed that absolute presenteeism demonstrated a positive correlation with *Bifidobacterium* (*rho* = 0.15, *p* < 0.05), ILA, phenyl lactic acid (PLA), hydroxyphenyllactic acid (HPLA), and homocysteine (*rho* = 0.15, 0.17, 0.17, and 0.16, *p*  < 0.05) and negatively correlated with urocanic acid (*rho* = −0.17, *p*  < 0.05) (Figure 5a). In contrast, the well-being score showed a positive correlation with *Fusicatenibacter*, acetic acid, butyric acid, and propionic acid (*rho*  = 0.16, 0.15, 0.14, and 0.16, respectively; *p* < 0.05) (Figure 5b).

**Figure 6.**
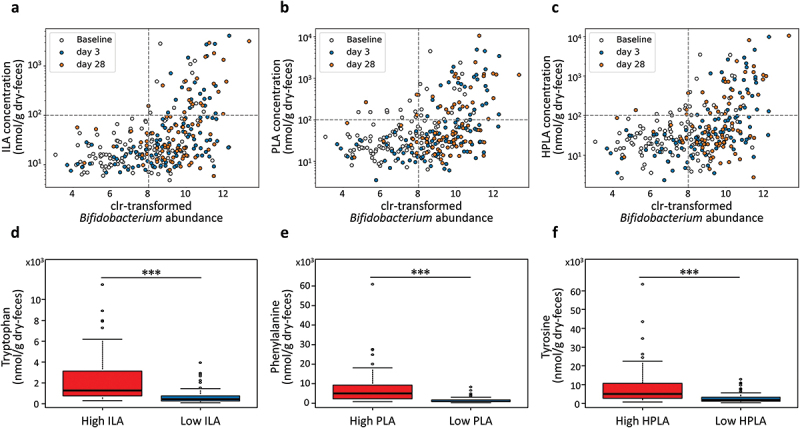
Interplay between *Bifidobacterium*, aromatic lactic acids (ALA), and aromatic amino acids concentrations. The scatter plots illustrate the relationship between *Bifidobacterium* and the concentrations of (a) indole lactic acid, (b) phenyl lactic acid, and (c) hydroxyphenyllactic acid. The color of the dots corresponds to baseline (white), day 3 (blue), and day 28 (orange). The boxplots depict the comparison of (d) tryptophan, (e) phenylalanine, and (f) tyrosine concentrations between the high-ala group (ALA’s concentration >100 nmol/g dry-feces) and the low-ala group (ALA’s concentration ≦ 100 nmol/g dry-feces) among *Bifidobacterium*-enriched samples (clr-transformed abundance > 8). Differences were compared using the Mann – Whitney U test (***p < 0.001).

In particular, *Bifidobacterium* drew our attention because of its dynamic changes during the intervention and its positive correlation with ILA, PLA, and HPLA (*rho* = 0.45, 0.35, and 0.36, respectively; *p* < 0.05). Despite the high abundance of *Bifidobacterium*, individual differences were observed in the concentrations of these ALAs (Figures 6a,b,c). To identify other factors influencing the concentration of ALAs, we focused on aromatic amino acids (AAAs) such as tryptophan, phenylalanine, and tyrosine, which are precursors of ALAs. We compared the concentrations of AAAs between samples with a high concentration of ALAs (high group) and those with a low concentration (low group) in samples with a high amount of *Bifidobacterium* (clr-transformed abundance > 8). The high ALAs group exhibited a higher concentration of AAAs in feces than the low-ALA group (Mann – Whitney U test, *p* < 0.001) (Figures 6d,e,f). Additionally, we conducted a partial correlation analysis between ALAs, *Bifidobacterium*, and AAAs. Both *Bifidobacterium* and AAAs were significantly correlated with ALA concentration, even when controlling for the influence of the other variable (*p* < 0.001) (Supplementary Table S9).

## Discussion

Recently, probiotics and prebiotics have been identified as potent modulators. They regulate the gut microbiota and stimulate the generation of beneficial metabolites such as short-chain fatty acids (SCFA) and ALAs.^29^ Consequently, they positively influence host health by promoting intestinal peristalsis,^11^ enhancing intestinal barrier function,^24,40^ and modulating the intestinal immune system.^22,25^ The gut microbiota affects several brain activities, including cognition and mentality^10,31^ via BGA, implying that modulation of these functions by probiotics and prebiotics could potentially improve QOL. Therefore, in this study, we examined the effects of a synbiotic dairy product (a combination of *B. longum* BB536 and lactulose) on host health and QOL, including its association with gut microbiota and metabolites.

The administration of synbiotic yogurt led to an increase in stool frequency and volume, BSFS score, absolute and relative presenteeism scores, and well-being score. The constituents of this synbiotic yogurt, *B. longum* BB536 and lactulose, have been recognized for their ability to improve stool frequency,^34,35,44^ volume,^35^ and form.^20,21^ These findings are consistent with those of previous studies. Furthermore, the trial revealed enhancements in psychological health and workplace performance. Recent studies have reported positive effects of probiotics and prebiotics on emotional, cognitive, systemic, and neural metrics.^38^ The statistically significant increase in the well-being score may potentially be attributed to the synbiotics *B. longum* BB536 and lactulose. Although some studies have reported that probiotic lactic acid bacteria improve work performance,^16,19,39^ data on the gut environment necessary to predict their mechanism of action are limited. Additionally, no reports are available on the effects of lactulose or bifidobacteria on presenteeism in healthy individuals.

In this study, we observed a positive correlation between absolute presenteeism and stool frequency. Patients with chronic constipation exhibit lower work productivity,^43^ suggesting that the intake of synbiotic products normalizes the individual’s stool frequency, leading to an increase in absolute presenteeism.

In this trial, we showed that the administration of synbiotic yogurt induced alterations in the gut microbiota and gut metabolites. *Bifidobacterium* exhibited the most significant increase in abundance. This outcome is attributable to the effect of lactulose, a prebiotic that promotes the growth of *Bifidobacterium* .^3,8,32,33^ In addition, we noted an increase in several bacteria, including butyric acid-producing bacteria, such as *Anaerobutyricum*, *Gemmiger*_A_73129, *Anaerostipes*, and *Faecalibacterium*. This observation may be attributed to cross-feeding with gut bacteria, such as bifidobacteria,^14,17,46^ and the elevation of essential nutrients for bacterial growth, such as amino acids,^8,42^ induced by the administration of synbiotic yogurt. The levels of various amino acids markedly increased post-intervention. The amino acids utilized by gut bacteria are converted into various bioactive substances, such as the ALAs ILA, PLA, and HPLA. ALAs are synthesized from AAAs and act as ligands for AhR and HCA3, which regulate immunity^22^ and metabolism.^9^ ILA significantly increased on day 28 compared to day 0. ILA is involved in the alleviation of intestinal inflammation,^48^ inhibition of infection,^54^ improvement of the intestinal barrier function,^47^ and regulation of immune responses during infancy.^22^ Synbiotic yogurt enhanced the production of these beneficial substances.

Post-intervention, metabolites in the nucleic acid group significantly increased. Although the function of nucleic acids in the large intestine is largely unknown, adenine and adenosine, which increased in this study, have been reported to exhibit antimicrobial activity against antibiotic-resistant pathogens, such as *Salmonella typhimurium*, *Staphylococcus aureus*, *Escherichia coli*, and *Klebsiella pneumonia*.^6^ However, because these bacterial species were present in relatively low quantities (average relative abundance 0%–0.27%) in this study, they may not have been identified as significantly altered microbial groups. Nonetheless, an increase in these nucleic acids could potentially contribute to the overall improvement of the gut microbiota by exerting antimicrobial effects on a broader range of bacterial species.

The well-being score exhibited a significant positive correlation with *Fusicatenibacter* and SCFA levels. Although *Fusicatenibacter* demonstrated a significant correlation with acetic acid and propionic acid (*rho*  = 0.24 and 0.17, *p* < 0.05), the causality remains uncertain because of the lack of detailed reports indicating that *Fusicatenibacter* produces these SCFAs. These SCFAs, recognized as the principal mediators of BGA, can suppress inflammation by enhancing the intestinal barrier function.^24^ Liu et al. suggested that inflammation originating in the intestine could be transmitted to the brain, potentially leading to psychological distress.^23^ Furthermore, SCFAs protect the central nervous system from toxic substances and peripheral inflammatory cytokines.^5^ Therefore, these SCFA functions could have contributed to the improvement in well-being score. Despite the expansion of several SCFA-producing bacteria, we did not observe a significant increase in SCFAs. However, it is possible that SCFAs, once absorbed into the body,^26^ contributed to the increase in well-being score. Further investigations such as blood metabolome analyses are warranted to elucidate the underlying mechanisms.

In contrast, ILA, PLA, and HPLA showed positive correlations with absolute presenteeism. PLA is an agonist for HCA3 and ILA acts as an agonist for both HCA3 and AhR.^22,36^ ALAs enhance the immune system and BGA.^1,22,30^ ILA induces NGF-mediated neurite outgrowth in a dose-dependent manner through AhR,^51^ and promotes the clearance of Aβ peptides by activating the AhR signaling pathway in microglia and astrocytes.^18^ These findings suggest that ILA plays a pivotal role in preserving neuronal and brain integrity by stimulating AhR. In addition, AhR activation has been reported to induce adult hippocampal neurogenesis, which contributes to learning and memory^49^ and improves behavior in model organisms.^56^ ALA-induced AhR activation may affect higher brain functions, such as learning and memory, potentially leading to an increase in absolute presenteeism.

In addition, certain bifidobacteria possess high ALA production capacity.^22,37^ In this trial, ILA, PLA, and HPLA also demonstrated a positive correlation with *Bifidobacterium*, suggesting that these ALAs were produced by *Bifidobacterium*. However, some samples exhibited low ALAs concentrations despite an abundance of *Bifidobacterium*. These samples contained low concentrations of AAAs (tryptophan, phenylalanine, and tyrosine). This finding suggests that the co-ingestion of *Bifidobacterium* with amino acids could be more effective in increasing the production of ALAs than consuming *Bifidobacterium* alone. Furthermore, yogurt, rich in amino acids, can positively contribute to ALA production. To further explore this relationship, we conducted a partial correlation analysis between ALAs, *Bifidobacterium*, and AAAs. Both *Bifidobacterium* and AAAs were significantly correlated with ALA concentration, even when controlling for the influence of the other variable (*p* < 0.001) (Supplementary Table S9). This indicates that both *Bifidobacterium* and AAAs are not confounding factors for each other and that the presence of both contributes to the increase in ALA levels. Therefore, further investigation into their individual roles is warranted to better understand the complex interactions within the gut microbiome and their impact on metabolic pathways.

In this study, we requested 200 participants to provide fecal samples thrice. However, less than half of these samples could not be analyzed because of inadequate drying. Most of the inadequately dried samples were collected after the intake of the synbiotic yogurt, with a few issues observed in the samples collected before intake. This suggests that the increase in stool frequency and volume, as well as the softening of the stool owing to the intake of synbiotic yogurt, may have inadvertently led the participants to collect more samples than before the intake. Therefore, more thorough instructions for participants during sampling and innovative ways to limit the number of fecal samples collected using the kit are needed, which will be a challenge that needs to be addressed in future studies.

This study has several limitations. First, this was an open-label, single-group, exploratory study. To evaluate its effectiveness, a double-blind randomized placebo-controlled trial is necessary. Second, the QOL was subjectively measured using questionnaires. An evaluation using objective measures, such as an electroencephalogram, is desirable. Third, further studies investigating the underlying molecular mechanisms are necessary to clarify the relationship between gut bacteria and QOL improvement. Fourth, this study did not consider the dietary habits of participants. Therefore, to confirm whether the metabolites detected in feces are indeed derived from gut bacteria, a trial that eliminates dietary influences is required. Despite these limitations, this study provides insights into the potential health benefits of synbiotic yogurt containing *B. longum* BB536 and lactulose.

In conclusion, our study indicates that the synbiotic combination of *B. longum* BB536 and lactulose could potentially enhance defecation status, QOL, and the gut environment. We observed that the production of ALAs by *Bifidobacterium* could potentially improve absolute presenteeism and that both the abundance of *Bifidobacterium* and the presence of AAAs are pivotal for the production of ALAs. These findings suggest that synbiotics containing *B. longum* BB536 and lactulose can ameliorate the gut environment and improve host well-being. Despite the limitations of this study, such as its open-label, single-group design, and subjective measurement of QOL, it provides valuable insights into the potential health benefits of synbiotic yogurt containing *B. longum* BB536 and lactulose. Future research is needed to further elucidate these relationships and validate these findings in larger and more diverse populations.

## Supplementary Material

Figure_S2.tif

Supplementary_table_v2 clean.xlsx

Figure_S1.tif

Figure_S3.tif

## Data Availability

The data presented in this study can be found in this published article.
