# Supplementary figures and images for: Synbiotic combination of Bifidobacterium longum BB536 and lactulose improves the presenteeism of healthy adults associated with aromatic lactic acids - A single-arm, open-label study
Source: Gut Microbes Rep. 2025 Apr 10;2(1):2490092. doi: 10.1080/29933935.2025.2490092 (PMC12940110; doi:10.1080/29933935.2025.2490092)

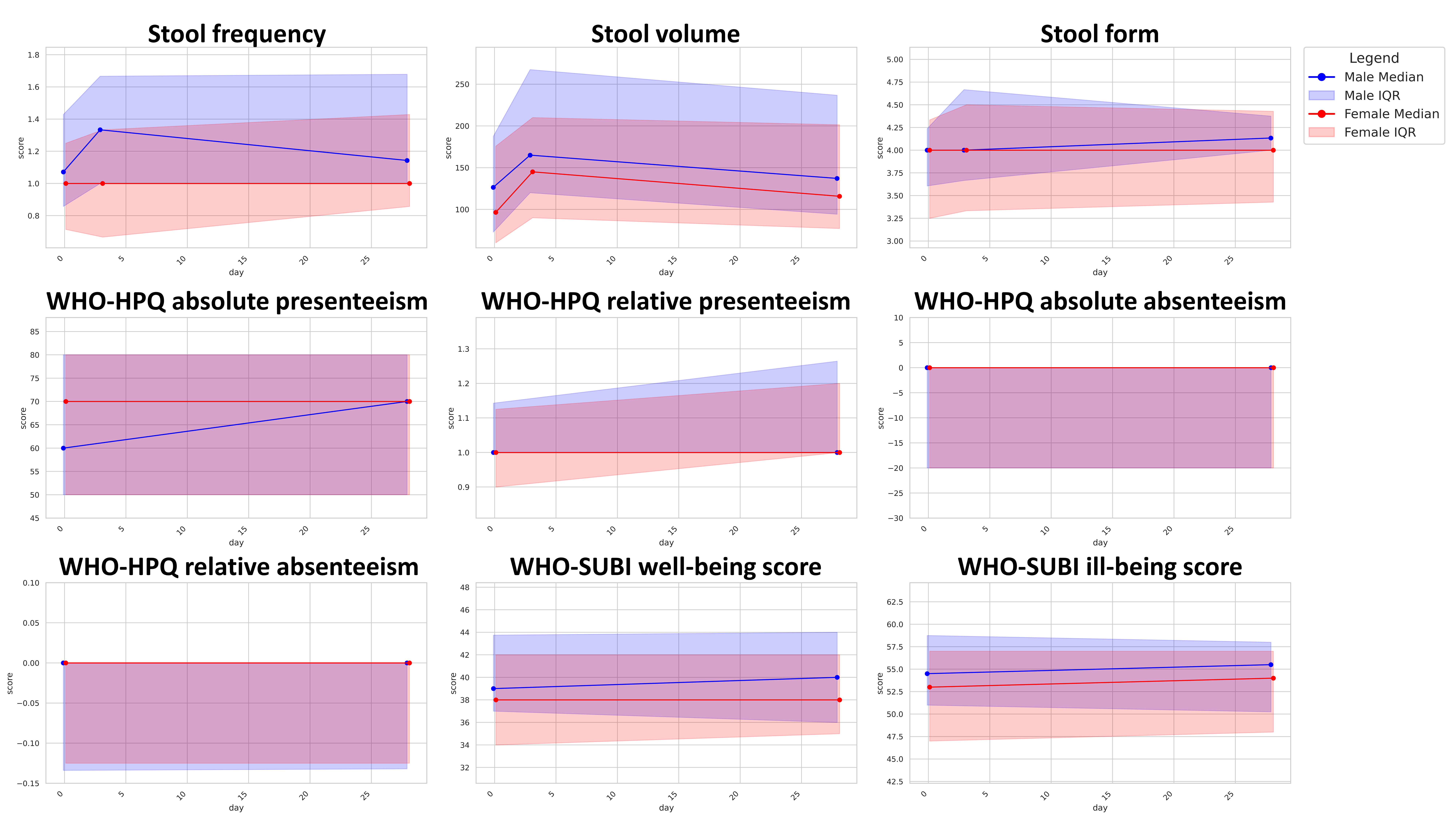

Supplement: Figure_S2.tif [file KGMR_A_2490092_SM7576.tif]

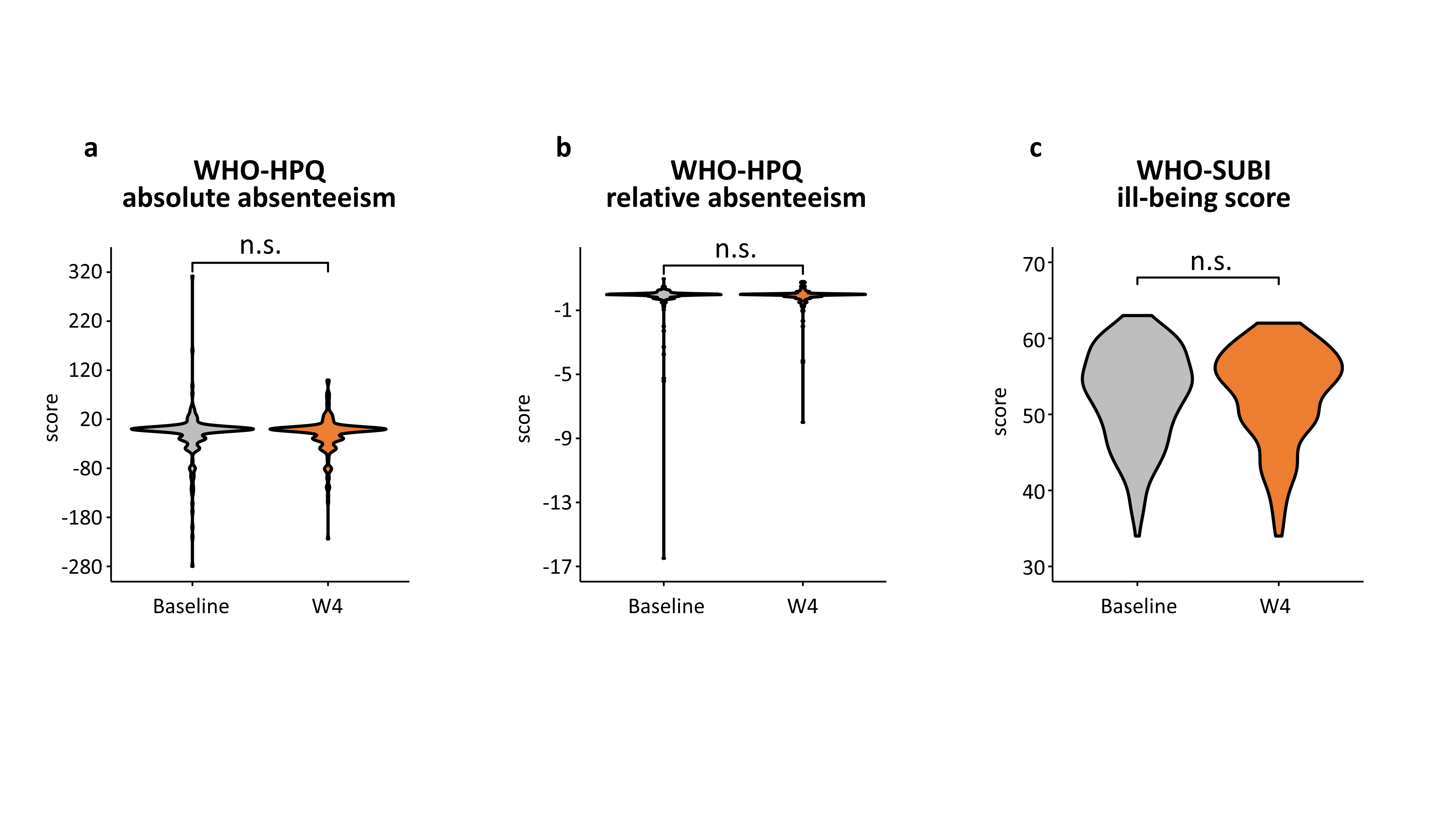

Supplement: Figure_S1.tif [file KGMR_A_2490092_SM7574.tif]

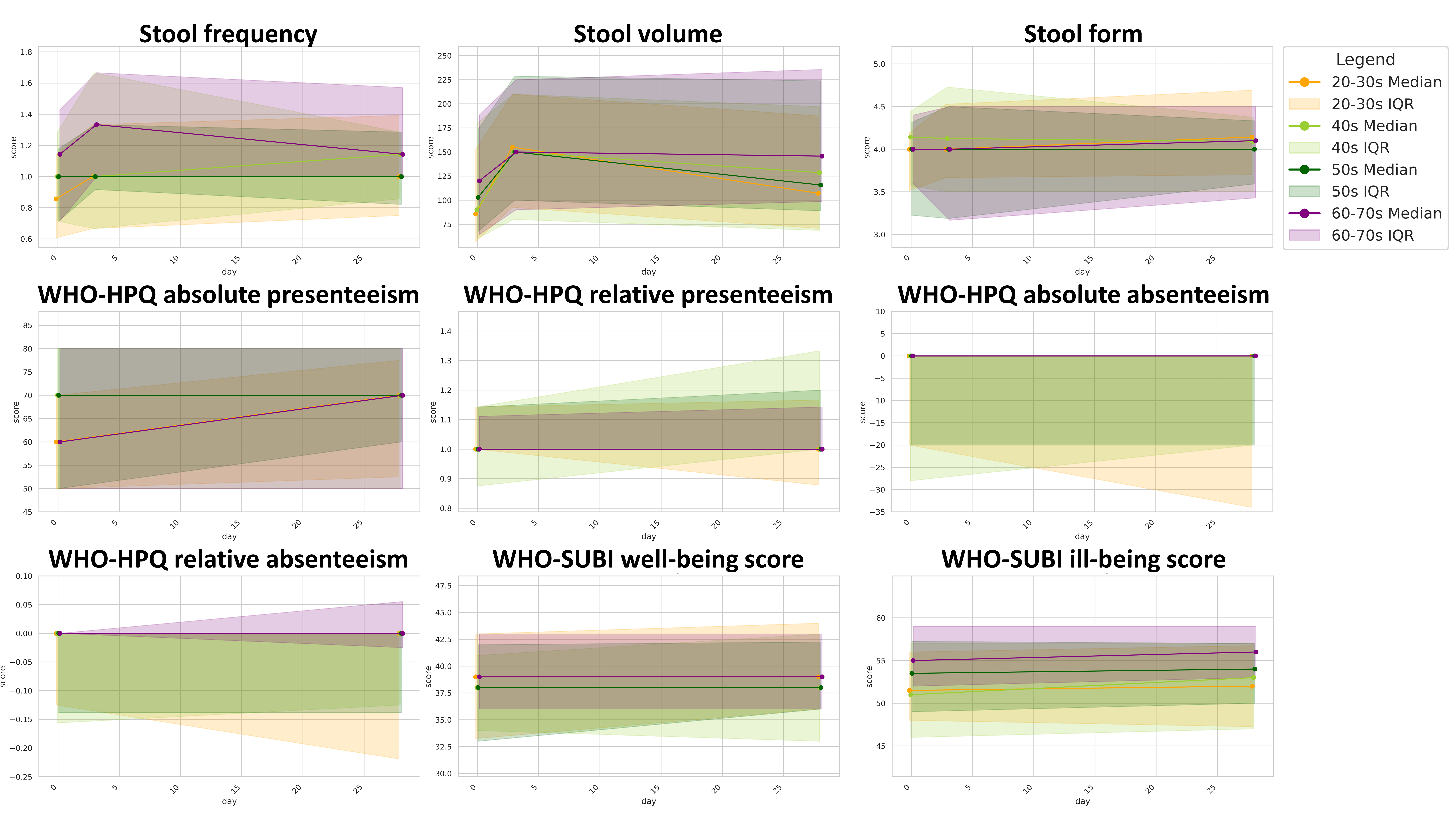

Supplement: Figure_S3.tif [file KGMR_A_2490092_SM7573.tif]
